# A Parent-of-Origin Effect Impacts the Phenotype in Low Penetrance Retinoblastoma Families Segregating the c.1981C>T/p.Arg661Trp Mutation of *RB1*

**DOI:** 10.1371/journal.pgen.1005888

**Published:** 2016-02-29

**Authors:** Philippine Eloy, Catherine Dehainault, Meriem Sefta, Isabelle Aerts, François Doz, Nathalie Cassoux, Livia Lumbroso le Rouic, Dominique Stoppa-Lyonnet, François Radvanyi, Gaël A. Millot, Marion Gauthier-Villars, Claude Houdayer

**Affiliations:** 1 Service de Génétique, Institut Curie, Paris, France; 2 CNRS UMR144, centre de recherche de l'Institut Curie, Paris, France; 3 Département d'oncologie pédiatrique, adolescents jeunes adultes, Institut Curie, Paris, France; 4 Université Paris Descartes, Sorbonne Paris Cité, Paris, France; 5 Département d’oncologie chirurgicale, service d’Ophtalmologie, Institut Curie, Paris, France; 6 INSERM U830, centre de recherche de l'Institut Curie, Paris, France; 7 Institut Curie, PSL Research University, Paris, France; 8 CNRS UMR 3244, Paris, France; 9 Sorbonne Universités, UPMC Univ Paris 06, Paris, France; Baylor College of Medicine, UNITED STATES

## Abstract

Retinoblastoma (Rb), the most common pediatric intraocular neoplasm, results from inactivation of both alleles of the *RB1* tumor suppressor gene. The second allele is most commonly lost, as demonstrated by loss of heterozygosity studies. *RB1* germline carriers usually develop bilateral tumors, but some Rb families display low penetrance and variable expressivity. In order to decipher the underlying mechanisms, 23 unrelated low penetrance pedigrees segregating the common c.1981C>T/p.Arg661Trp mutation and other low penetrance mutations were studied. In families segregating the c.1981C>T mutation, we demonstrated, for the first time, a correlation between the gender of the transmitting carrier and penetrance, as evidenced by Fisher’s exact test: the probability of being unaffected is 90.3% and 32.5% when the mutation is inherited from the mother and the father, respectively (p-value = 7.10^−7^). Interestingly, a similar correlation was observed in families segregating other low penetrance alleles. Consequently, we investigated the putative involvement of an imprinted, modifier gene in low penetrance Rb. We first ruled out a *MED4*-driven mechanism by *MED4* methylation and expression analyses. We then focused on the differentially methylated CpG85 island located in intron 2 of *RB1* and showing parent-of-origin-specific DNA methylation. This differential methylation promotes expression of the maternal c.1981C>T allele. We propose that the maternally inherited c.1981C>T/p.Arg661Trp allele retains sufficient tumor suppressor activity to prevent retinoblastoma development. In contrast, when the mutation is paternally transmitted, the low residual activity would mimic a null mutation and subsequently lead to retinoblastoma. This implies that the c.1981C>T mutation is not deleterious *per se* but needs to be destabilized in order to reach pRb haploinsufficiency and initiate tumorigenesis. We suggest that this phenomenon might be a general mechanism to explain phenotypic differences in low penetrance Rb families.

## Author Summary

Complex genotype-phenotype correlations lead to clinically and emotionally difficult situations. Improved understanding of these correlations is of utmost importance in medical genetics. Low penetrance retinoblastoma families segregating the c.1981C>T / p.Arg661Trp mutation are a good model as germline carriers develop bilateral, unilateral retinoblastoma, benign retinomas or remain unaffected. The c.1981C>T mutation results in a mutant pRb protein that is partially inactivated which may explain the reduced severity observed. However it is still unclear why this would be the case in some family members and not in others. We have demonstrated a parent-of -origin effect in c.1981C>T / p.Arg661Trp pedigrees and have concluded that overexpressed maternally inherited p.Arg661Trp alleles retain sufficient tumor suppressor activity to prevent Rb development. This might be a general phenomenon driving low penetrance retinoblastoma. Our findings shed light on genotype-phenotype correlations in low penetrance retinoblastoma and are of special relevance for genetic counselling.

## Introduction

Retinoblastoma (Rb) is the most common pediatric intraocular neoplasm and occurs in 1 of every 15,000 births. It results from the biallelic inactivation of the *RB1* tumor suppressor gene, located on 13q14 [1]. *RB1* encodes the nuclear phosphoprotein pRB, which plays a prominent role during the G1/S phase transition[2].

In tumors, both *RB1* alleles can be inactivated via diverse mechanisms including point mutations, large rearrangements, promoter hypermethylation and, most frequently, loss of the second allele demonstrated by loss of heterozygosity studies. In non-hereditary retinoblastoma, both *RB1* mutations are somatic and occur in the same retinal cell that develops into a tumor. In contrast, in hereditary retinoblastoma, germline mutation of one allele is associated with predisposition to Rb, while the second mutation on the other allele is somatic, usually acquired during early childhood. Non-hereditary retinoblastomas are usually unilateral (one eye affected) with a median age at diagnosis of 2 years, whereas hereditary cases are usually bilateral (both eyes affected) with a median age at diagnosis of 1 year and an increased risk for second tumors.

Familial hereditary Rb is defined as two or more carriers of an *RB1* germline gene mutation in a family and represents 10% of all retinoblastomas. Predisposition to Rb is transmitted as an autosomal dominant trait with almost complete penetrance (over 90%) and germline carriers usually develop bilateral or multifocal tumors. However, some Rb families display low penetrance (unaffected carriers) and variable expressivity (carriers develop bilateral, unilateral Rb or even benign retinomas). Deciphering the mechanisms underlying low penetrance (LP) Rb is of utmost importance, as it will impact the clinical management of these families and furthers our understanding of Rb as a disease.

The well-known c.1981C>T / p.Arg661Trp low penetrance mutation in exon 20 of *RB1* results in a mutant pRb that is partially inactivated [3,4] which may explain the reduced severity observed. However, why this would be the case in some family members and not in others remains unclear. Based on the collection of large families, we have demonstrated, that in the context of c.1981C>T/p.Arg661Trp low penetrance, a parent-of-origin effect impacts on Rb phenotype. When the mutation is inherited from the paternal side, offspring are retinoblastoma-prone. In contrast, when the mutation is inherited from the maternal side, offspring mostly remain unaffected. Based on these observations, the involvement of a putative modifier, imprinted gene should be considered. Two alternative hypotheses were tested. Firstly, we searched for a possible involvement of the *MED4* gene, located in the flanking centromeric region of *RB1*, as we have recently demonstrated that *MED4* expression is required for Rb development [5]. We postulated maternal imprinting for *MED4*, which results in monoallelic expression from the paternal allele. As a result, when the *RB1* c.1981C>T/p.Arg661Trp mutation is inherited from the mother, loss of the contralateral paternal allele in the tumor would switch off *MED4* expression and prevent retinoblastoma development in the context of a low penetrance mutation. Secondly, we focused on a differentially methylated CpG island showing parent-of-origin-specific DNA methylation at the *RB1* gene and located in *RB1* intron 2 (called CpG85 hereafter) [6,7]. Differential methylation of CpG85 skews *RB1* expression in favor of the maternal allele [6]. Our results on a series of germline, tumor DNAs and RNAs did not support any involvement of *MED4* in the low penetrance phenotype, but confirmed the differentially methylated status of *RB1* CpG85. It was therefore concluded that overexpressed maternally inherited p.Arg661Trp alleles retain sufficient tumor suppressor activity to prevent Rb development. On the other hand, when the mutation is paternally transmitted, the low residual activity would mimic a null mutation, leading to haploinsufficiency and Rb development.

## Results

### Description of the families

We reviewed the records of 49 pedigrees from Institut Curie with a family history of Rb. Thirty-four of these families segregated high penetrance mutations and 15 families segregated low penetrance mutations. Eight low penetrance families derived from the literature were also found by PubMed search and were added to the study (Table 1). All first generation carriers were excluded to avoid any bias in DER calculation (Disease Eye Ratio, see “Patients and Methods” section).

**Table 1:** Description of low penetrance families. DER: disease-eye ratio (see text for details). Nomenclature follows HGVS rules using the reference sequence NM_000321.2. Previously published families are indicated. Pedigrees F6, F7, F16, F17, F20-22 were from our series and have been published in part (see text for details). Families F14 and F15 were removed from statistical analysis (see text for details).

Family	Mutation description	Expected consequence	Number affected	Total number of carriers	DER	Comments
**F1**[8]	c.1981C>T	p.Arg661Trp	5	6	1	4 Unilateral Rb
						1 Bilateral Rb
**F2**[8]	c.1981C>T	p.Arg661Trp	3	5	0.6	3 Unilateral Rb
**F3**[9]	c.1981C>T	p.Arg661Trp	7	18	0.56	3 Bilateral Rb
						4 Unilateral Rb
**F4**[10]	c.1981C>T	p.Arg661Trp	6	10	0.8	2 Bilateral Rb
						2 Unilateral Rb
						2 retinomas
**F5**	c.1981C>T	p.Arg661Trp	2	11	0.18	2 Unilateral Rb
**F6**[11]	c.1981C>T	p.Arg661Trp	4	8	1	4 Bilateral Rb
**F7**[11]	c.1981C>T	p.Arg661Trp	5	18	0.33	4 Unilateral Rb
						1 Bilateral Rb
**F8**	c.1981C>T	p.Arg661Trp	1	2	0.5	1 Unilateral Rb
**F9**	c.1981C>T	p.Arg661Trp	1	3	0.33	1 Unilateral Rb
**F10**	c.1981C>T	p.Arg661Trp	1	3	0.67	1 Bilateral Rb
**F11**[12]	c.1960G>C	p.Val654Leu	7	16	0.44	7 Unilateral Rb
**F12**	c.1960G>A	p.Val654Met	1	4	0.5	1 Bilateral Rb
**F13**	c.10A>T	p.Lys4*	1	3	0.33	1 Unilateral Rb
**F14**[13]	c.607+1G>T	Exon 6 skipped	13	25	0.84	5 Unilateral Rb
						8 Bilateral Rb
**F15**[13]	c.607+1G>T	Exon 6 skipped	3	10	0.4	2 Unilateral Rb
						1 Bilateral Rb
**F16**[11]	c.607+1G>T	Exon 6 skipped	2	5	0.4	2 Unilateral Rb
**F17**[11]	c.607+1G>T	Exon 6 skipped	3	5	1	1 Unilateral Rb
						2 Bilateral Rb
**F18**	c.1696-2A>G		2	4	0.5	2 Unilateral Rb
**F19**[14]	c.1331A>G	Exon 13 skipped	2	8	0.25	2 Unilateral Rb
**F20**[11]	c.45_79dup	p.Pro27Leufs*50	1	6	0.17	1 Unilateral Rb
**F21**[11]	c.1422-2A>G	Exon 16 skipped	3	4	1	2 Unilateral Rb
						1 Bilateral Rb
**F22**[11]	c.-193T>G	Promoter	2	3	1	1 Unilateral Rb
						1 Bilateral Rb
**F23**	c.19del	p.Arg7Glufs*58	1	5	0.2	1 Unilateral Rb
**F24**[15]	c.43_65dup	p.Pro23Leufs*50	4	10	0.4	3 Unilateral Rb
						1 retinoma
**F25**[16]	c.862-10T>C	Exon 9 skipped	4	9	0.55	3 Unilateral Rb
						1 Bilateral Rb

#### High penetrance families

Unilateral Rb and bilateral Rb were identified in 7 (3 males and 4 females) and 71 patients (34 males and 37 females), respectively. Mean DER was 1.94.

#### p.Arg661Trp low penetrance families

**Fig 1:**
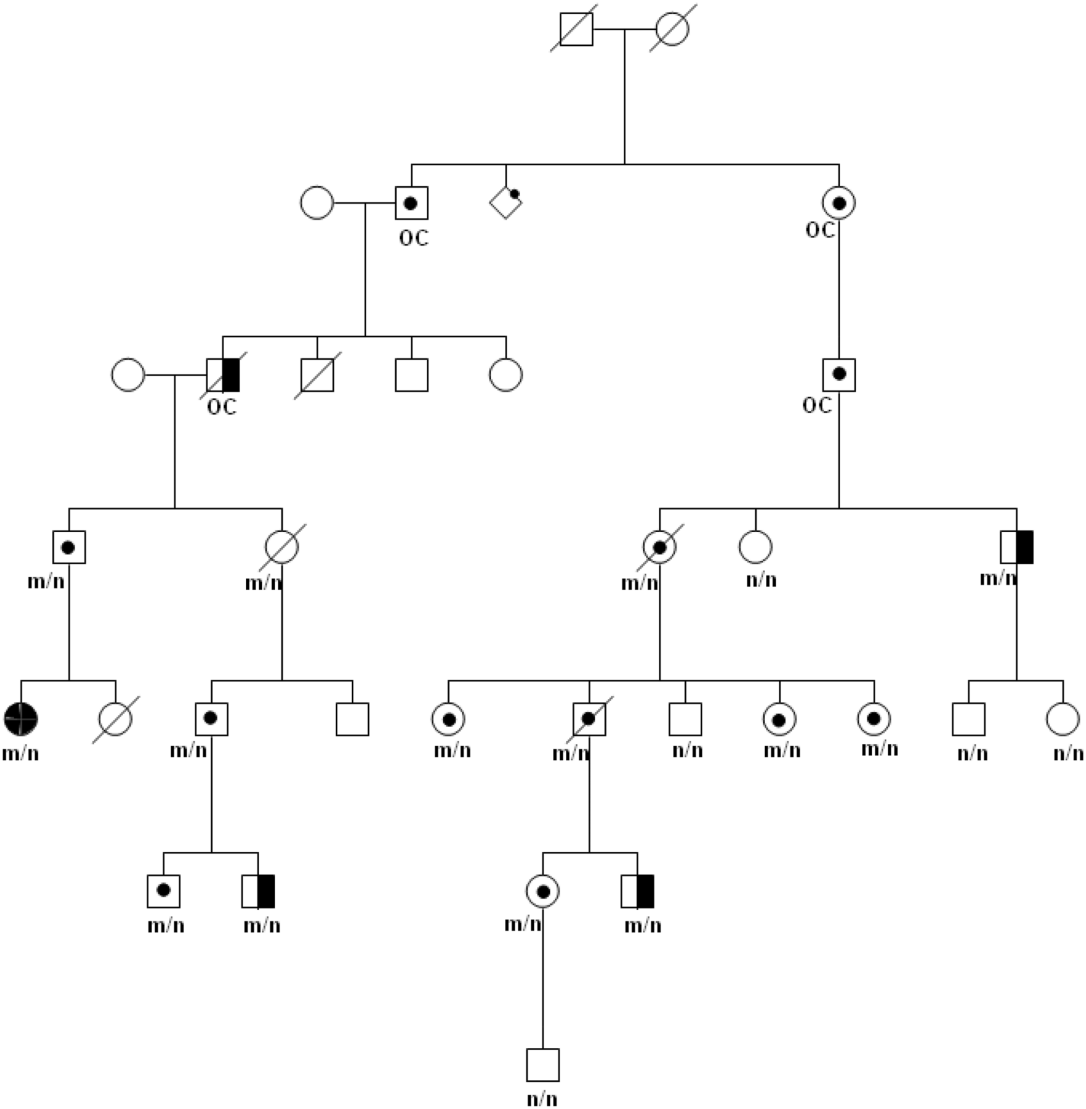
Family F7 segregating the *RB1* c.1981C>T/p.Arg661Trp mutation. Genotype is provided for tested members as m/n for heterozygous carriers and n/n for homozygous wild-type. OC indicates obligate carriers. Blackened symbols: bilateral Rb; half-blackened symbols: unilateral Rb; dotted symbols: unaffected carriers; dashed symbols: deceased.

Six families were derived from Institut Curie and 4 families were derived from the literature, corresponding to 85 germline carriers: 49 males and 36 females. Unilateral Rb was identified in 21 patients (12 males and 9 females), bilateral Rb was identified in 12 patients (9 males and 3 females) and retinoma was identified in 2 patients. The remaining 50 carriers were unaffected. Mean DER was 0.60. One pedigree segregating the c.1981C>T/p.Arg661Trp mutation is shown (Fig 1).

#### Other low penetrance families

We found 9 families from Institut Curie and 6 families from the literature segregating a non- c.1981C>T/p.Arg661Trp low penetrance *RB1* mutation (*i*.*e*. c.-193T>G, c.10A>T, c.19del, c.43_65dup, c.45_79dup, c.607+1G>T, c.862-10T>C, c.1331A>G, c.1422-2A>G, c.1960G>A, c.1960G>C), corresponding to 127 carriers of a germline mutation. Unilateral Rb and bilateral Rb were identified in 34 and 15 patients, respectively, and 68 carriers were unaffected. Mean DER was 0.53

### Statistical analysis

The parental origin of the c.1981C>T/p.Arg661Trp mutant allele was documented in 71 of the 85 carriers. In this series of 71 carriers, 31 and 40 received the mutant allele from their mother and father, respectively. Twenty-eight carriers who received the mutant allele from their mother remained unaffected (28/31, 90.3%), and only 3 developed Rb (3/31, 9.7%). In contrast, 13 carriers who received the mutant allele from their father remained unaffected (13/40, 32.5%) and 27 developed Rb (27/40, 67.5%). Consequently, inheriting the c.1981C>T/p.Arg661Trp mutation from the maternal side significantly prevented Rb development (p-value = 7.10^−7^, Fisher’s exact test). In other words, the probability of being unaffected when the mutation is inherited from the maternal side is 90.3% versus only 32.5% when the mutation is inherited from the paternal side.

We then looked for a similar disequilibrium in families segregating non-p.Arg661Trp low penetrance mutant alleles (see Table 1). To avoid any bias, families F14 and F15 segregating the c.607+1 G>T mutation were excluded from analysis since a parent of origin effect was previously described [13]. The parental origin of the mutant alleles was documented in 58 of the 82 remaining carriers. Seventeen carriers received the mutation from their mother and 41 received the mutation from their father. Thirteen carriers who received the mutant allele from their mother were unaffected (13/17, 76.4%) and 4 developed Rb (4/17, 23.6%). Eighteen carriers who received the mutation from their father were unaffected (18/41, 43.9%) and 23 developed Rb (23/41, 56.1%). Fisher’s exact test demonstrated a disequilibrium between the gender of the transmitting carrier parent and penetrance (p-value = 0.041). Lastly, families segregating high penetrance mutations displayed no such correlation, as all 54 mutation carriers of known parental origin developed retinoblastoma, regardless of the gender of the transmitting carrier. As previously described, no preferential transmission of mutant or normal alleles from carrier fathers or mothers was observed [17]. These results unambiguously demonstrate that, in the context of low penetrance Rb, a parent-of-origin effect impacts on Rb phenotype.

### *RB1* CpG85 methylation analyses

#### Blood samples

To determine whether *RB1* CpG85 is differentially methylated in a parent-of-origin-specific manner, we studied the methylation pattern of 9 CpG dinucleotides within the CpG85 island using bisulfite treatment and pyrosequencing in DNAs extracted from blood. For all non-deleted *RB1* samples (i.e. with 2 *RB1* alleles), the C-to-T ratio at the CpG dinucleotides studied was close to 1:1, indicating 50% methylation at CpG85 (S1A Fig). We then studied 6 Rb patients with a large *RB1* deletion of known parental origin. All 3 patients with loss of the maternal *RB1* allele showed absence of methylation at CpG85 (S1B Fig). In contrast, CpG85 was fully methylated in all 3 patients with loss of the paternal *RB1* allele (S1C Fig). These results confirmed that *RB1* is imprinted and that CpG85 is specifically methylated on the maternal allele.

#### Tumor samples

**Fig 2:**
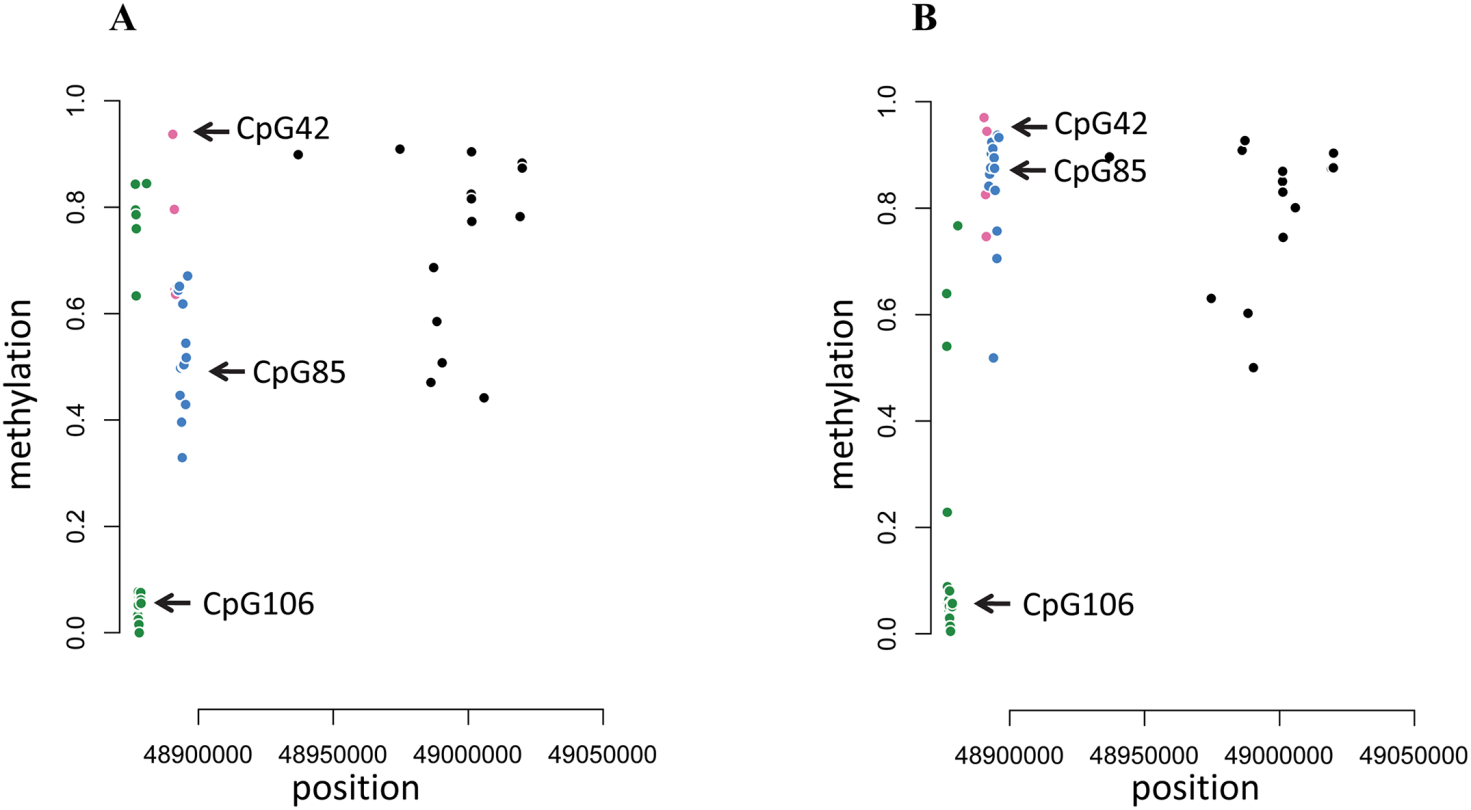
Methylation analyses of *RB1* CpG islands using methylation array. X axis represents the position on chromosome 13. Y axis represents overall methylation level. CpG106 localizing in *RB1* promoter is shown in green, CpG42 is shown in pink and CpG85 is shown in blue. For each sample, multiple CpGs are located within an island and each dot represents a single result. A: Normal retina. CpG85 showing approximately 50% of methylation. B: Tumor sample. CpG85 displaying a hypermethylated profile.

To assess a putative imprinting defect at CpG85, the methylation level of 3 CpG islands within *RB1* was analysed in 2 normal retinas and 45 tumors. CpG106 is located in the promoter region, while CpG85 and CpG42 are located in intron 2. In normal retina DNAs, CpG106 was hypomethylated, while a high DNA methylation level was observed for CpG42 (Fig 2A). CpG85 displayed approximately 50% methylation (Fig 2A), in agreement with genomic imprinting at this locus. Interestingly, CpG85 was fully methylated in all but 3 tumor DNA samples (Fig 2B). These results strongly suggest, for the first time, loss of imprinting at CpG85 locus in retinoblastoma.

#### *RB1* allelic imbalance

**Fig 3:**
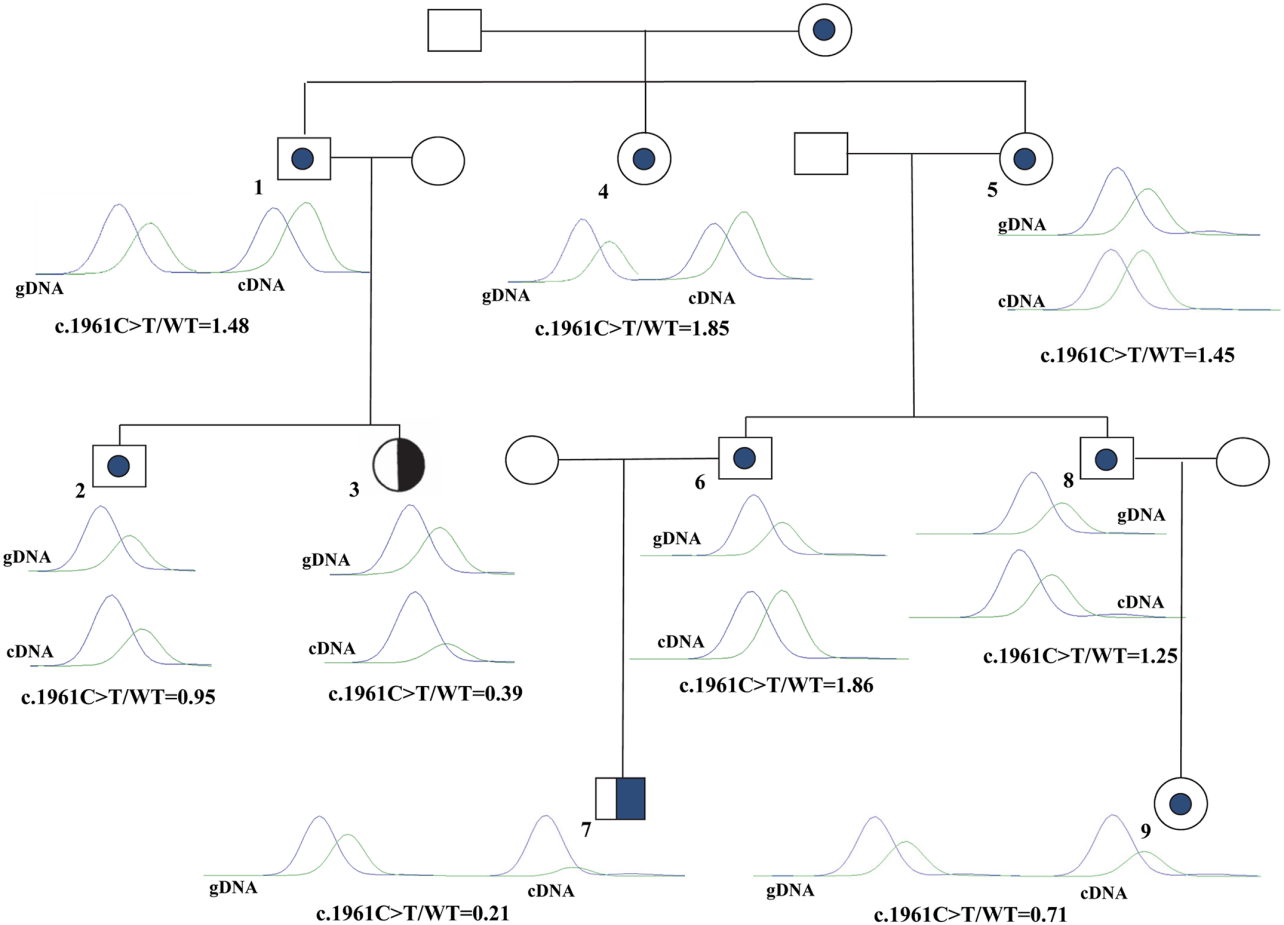
*RB1* allelic imbalance in family F5. The normalized SNaPshot cDNA ratio between the mutant and the wild type alleles are indicated below each carrier individual with corresponding SNaPshot results. The c.1981C>T/p.Arg661Trp mutant allele “T” is indicated in green and the wild type allele “C” is indicated in blue. Dotted symbols: unaffected carriers; half-blackened symbols: unilateral Rb.

To assess specific expression imbalance of *RB1* according to the sex of the transmitting parent, a quantitative SNaPshot assay targeting the c.1981C>T/p.Arg661Trp mutation was used within a series of 20 carriers including the low penetrance family F5. To avoid any bias due to a putative exon skipping, exon 20 inclusion was first confirmed by a dedicated RNA study (S2 Fig). Next, using the mutated allele as a marker, we found allelic imbalance in favour of the maternal allele in all 20 patients, albeit to different extend (Table 2 and Fig 3). Surprisingly, in family F5, unaffected carriers 1, 4, 5, 6 and 8 showed higher expression of the mutant allele whereas affected probands 3 and 7 showed higher expression of the wild type allele. Allelic ratio was close to equilibrium for the unaffected carrier 2 and in favour of the mutant allele for the unaffected carrier 9. A similar expression pattern was found in the other c.1981C>T carriers as all affected individuals showed a lower expression of the mutant allele (Table 2). These results confirmed the higher expression of the maternally transmitted *RB1* allele but raised questions about genotype-phenotype correlation as higher expression of the mutant allele and lack of penetrance appeared to be linked.

**Table 2:** Expression imbalance in 20 carriers of the c.1981C>T/p.Arg661Trp mutation. Transmission in family F5 is detailed Fig 3. First degree relatives are indicated for the other families. See text for ratio calculation. (*) See Fig 3.

Family	Patient	Carrier status	Parental origin of the c.1981C>T allele	Ratio c.1981C>T /WT
F5	1	Unaffected	Maternal*	1.48
F5	2	Unaffected	Paternal*	0.95
F5	3	Unilateral	Paternal*	0.39
F5	4	Unaffected	Maternal*	1.85
F5	5	Unaffected	Maternal*	1.45
F5	6	Unaffected	Maternal*	1.86
F5	7	Unilateral	Paternal*	0.21
F5	8	Unaffected	Maternal*	1.25
F5	6	Unaffected	Paternal*	0.71
F6	1	Bilateral	Paternal	0.69
F6	2	Unaffected	Paternal	0.46
F6	3	Bilateral	Paternal (son of F6-2)	0.40
F7	1	Unaffected	Maternal	2.27
F7	2	Unilateral	Paternal	0.69
F7	3	Bilateral	Paternal	0.62
F8	1	Unilateral	First generation carrier	0.38
F8	2	Unaffected	Maternal (daughter of F8-1)	1.43
F9	1	Unaffected	First generation carrier	0.50
F9	2	Unilateral	Paternal (daughter of F9-1)	0.85
F9	3	Unaffected	Paternal (daughter of F9-1)	0.91

#### *MED4* analyses

To determine whether *MED4* CpG53 is differentially methylated in a parent-of-origin-specific manner, we used bisulfite treatment and pyrosequencing on 24 DNA samples extracted from blood. CpG53 from all 24 samples showed fully unmethylated pattern. Similarly, the 45 tumor DNAs and normal retina analyzed by bisulfite treatment and methylation array were found to be unmethylated at the CpG53 locus (S3 Fig). These results excluded a parent-of-origin regulation of *MED4* via *MED4* promoter differential methylation.

To assess whether *MED4* is imbalanced in a parent-of-origin manner, we looked for monoallelic expression using the *MED4* rs41284209 SNP as a marker. We identified 9 patients who were heterozygous carriers of the rs41284209 SNP. No significant allelic disequilibrium was detected by Sanger sequencing and SNaPhot analyses (S4 and S5 Figs). Overall these results showed that both parental alleles contributed equally to *MED4* expression and ruled out *MED4* parental imprinting.

## Discussion

Deciphering the molecular basis of low penetrance retinoblastoma is of utmost importance for both researchers and clinicians, as it will shed light on retinoblastoma development, allow prognostic assessment in low penetrance families, and promote optimal genetic counseling and ophthalmological surveillance. In this study, we have identified, for the first time, a parent-of-origin effect in families segregating the c.1981C>T/p.Arg661Trp mutation. In these families, the probabilities of being unaffected for germline carriers were 90.3% and 32.5% when the mutation was inherited from the maternal and paternal side, respectively. Interestingly, a similar correlation was observed in families segregating other low penetrance alleles, albeit to a lesser extent: probabilities of being unaffected were 76.5% and 43.9% when the mutation was inherited from the maternal and paternal side, respectively. This finding echoes the maternal protective effect previously described in 2 families (F14 and F15 in this paper) in association with the c.607+1G>T low penetrance mutation [13]. Restoration of the maternal truncated transcript or mutation at an imprinted locus in *cis* were proposed to explain this observation. Our own results on a large number of pedigrees segregating a distinct low penetrance mutation rule out the first hypothesis, but support the second hypothesis.

We have recently shown that retinoblastoma *RB1* -/- cells cannot survive in the absence of *MED4*, both *in vitro* and in orthotopic xenograft models *in vivo*, therefore identifying *MED4* as a survival gene in retinoblastoma [5]. Consequently, we considered a *MED4*-driven general mechanism to explain low penetrance retinoblastoma. We postulated a parent-of-origin regulation of *MED4* that would be able to skew *MED4* expression in favor of the maternal allele. As a result, when the p.Arg661Trp mutation is inherited from the mother, loss of the contralateral paternal allele would dramatically decrease *MED4* expression and prevent retinoblastoma development in the context of a low penetrance mutation. However, methylation and expression studies both ruled out this mechanism to explain the parent-of-origin effect observed in p.Arg661Trp pedigrees.

A recent study demonstrated *RB1* imprinting by a differentially-methylated-region (DMR) at CpG85 in *RB1* intron 2. In humans, this DMR is methylated on the maternal allele and remains unmethylated on the paternal allele. Consequently, CpG85 acts as a weak promoter for an alternative, paternally expressed, *RB1* transcript (*RB1*-E2B) that competes with the main *RB1* transcript. This transcriptional interference skews *RB1* expression in favor of the maternal allele [6,18].

In line with this previous report, our SNAPshot analyses targeting the c.1981C>T/p.Arg661Trp mutation demonstrated higher expression of the maternal *RB1* allele. Our results also demonstrated that, when this mutation is inherited from the maternal side, offspring mostly remain unaffected. Although counter-intuitive, this means that a high level of the c.1981C>T/p.Arg661Trp mutant allele would protect from retinoblastoma. A plausible explanation lies in the residual biochemical properties of p.Arg661Trp mutants, which lack E2F pocket protein-binding activity but retain E2F-independent tumor suppressor function and the wild-type ability to partially suppress colony growth of RB(-) cells and induce parameters of cell differentiation [19]. More broadly, an E2F-independent paradigm of tumor suppression is being developed for *RB1*[20]. Lastly, a study showed that certain LP alleles (p.Arg661Trp included) retain greater functional activity than expected, which is why additional cooperating events are needed to block this residual activity [21]. The competing *RB1*-E2B transcript that lowers *RB1* regular transcript on the paternal allele might constitute this additional event in low penetrance Rb families. Consequently, when the father transmits the mutation, the residual pRb activity is too low to prevent the development of Rb in the cell. The low residual activity would mimic a null mutation, leading to genomic instability and Rb development. This also means that the c.1981C>T/p.Arg661Trp mutation is not deleterious *per se* but needs to be destabilized in order to reach pRb haploinsufficiency and initiate genomic instability and tumorigenesis [22,23]. Although our results on low penetrance families segregating other LP alleles reached borderline significance (p = 0.041), we propose this hypothesis as a general mechanism to explain disease occurrence in the context of low penetrance Rb.

Intriguingly, we have also reported, for the first time, a hypermethylated, deregulated *RB1* imprint in Rb tumors. Hypermethylation of CpG85 inhibits *RB1*-E2B transcription, therefore enhancing *RB1* main transcript expression. A plausible explanation would be that this loss of imprinting at the CpG85 locus might be used by tumor cells to attempt to increase the expression of pRB and thus restore its tumor suppressor activity.

Overall, we demonstrated that a parent-of-origin effect is involved in low penetrance Rb families segregating the c.1981C>T/p.Arg661Trp mutation of *RB1* and propose this phenomenon as a general mechanism to explain phenotypic differences in low penetrance Rb families.

## Materials and Methods

### Ethics statement

All patients have given written informed consent during genetic counselling sessions. The study was approved by the *Groupe Thématique Transverse (GTT) “retinoblastome”* of Institut Curie medical center (2013–2310).

### Patients

Institut Curie is the national referral center for retinoblastoma in France. Diagnosis of Rb is established on the basis of examinations by an ophthalmologist and histopathological criteria when treatment involves enucleation. All Rb patients are offered genetic counseling and *RB1* gene mutation analysis in constitutional and tumor DNA. When a germline mutation is found, molecular testing is extended to relatives. Individual written consent for genetic analysis was obtained from all participating patients or their legal guardians. The study was approved by our local ethic committee and retinoblastoma board.

In our series of 1,210 consecutively ascertained cases, we surveyed 49 pedigrees with a family history of Rb. Seven of the low penetrance families have been previously published in part[11]. We included family members for which the mutational status was ascertained by *RB1* analysis and obligate carriers when a DNA sample was not available. Relatives underwent routine fundus examination to look for the presence of retinomas (retinal scars). Since it has been described that retinoma develops after homozygous loss of *RB1*[24], individuals with retinoma were considered to be affected. Obligate carriers with normal fundus examination were considered to be non-penetrant or unaffected. Mutational mosaicism is known to explain the variable expressivity and penetrance in Rb patients[25]. Consequently, we excluded all first-generation carriers of a germline mutation displaying unilateral Rb or remaining unaffected, since retinal mosaicism could not be excluded in these patients. Clinical features included disease status (affected / unaffected) and diseased-eye ratio (DER). The DER is defined as the ratio of the sum of the eyes affected by tumors to the number of mutation carriers in a family. It provides a useful combination of penetrance and expressivity. Families with a DER ≥ 1.5 are considered to display complete penetrance. Families with a DER ≤ 1 are designated as LP[8].

### Statistical analysis

Fisher’s exact test was performed using R statistical software v3.0.2 on i) 10 p.Arg661Trp families (6 from our series and 4 from the literature [8,9,10]), ii) 13 non-p.Arg661Trp low penetrance families (9 from our series and 4 from the literature [11,12,14,15,16], iii) 34 high penetrance families from our series.

### *RB1* CpG85 and *MED4* CpG53 methylation analyses

#### Blood samples and pyrosequencing

Blood DNAs from 17 Rb patients (including 2 carriers of the p.Arg661Trp mutation and 6 carriers of a large deletion of known parental origin) and 2 controls underwent bisulfite conversion using the EZ DNA Methylation-Gold kit (Zymo research) according to the manufacturer’s instructions. Pyrosequencing primers were designed to cover the whole CpG85 island using the PyroMark Assay Design Software v1.0.6 (Qiagen). Bisulfite conversion was assessed by PCR amplification using converted DNA specific primers and agarose gel electrophoresis. Samples were prepared with the PyroMark Q96 Vacuum Workstation and pyrosequencing was performed on a PyroMark Q96 (Qiagen) according to the manufacturer’s instructions with subsequent analysis using the Analysis software package v2.5.7 (Qiagen).

#### Tumor samples and CGH methylation analyses

Tumor DNAs from 45 Rb patients (6 bilaterally and 39 unilaterally affected cases, respectively) and DNA samples from 2 normal retina were collected and hybridized on Infinium HumanMethylation 450 BeadChip arrays (Illumina, San Diego, CA). Prior to hybridization, DNA samples underwent bisulfite conversion using the EZ DNA Methylation Kit (Zymo Research). Four microliters of bisulfite-converted DNA were used for hybridization, following the Illumina Infinium HD Methylation protocol. Data were normalized using GenomeStudio (Illumina, Inc.) and R statistical software v3.0.2.

### Expression analyses

#### RNA extraction and RT PCR

Total RNAs were extracted from 200 μL frozen stabilized blood samples with the Nucleospin RNA Blood kit (Macherey-Nagel), according to the manufacturer’s instructions. Nagel). RNA quality was controlled using the NanoDrop 1000 Spectrophotometer (Thermo Scientific). Reverse transcription (RT) was performed with random hexamers using the RNA PCR core kit GeneAmp (Applied Biosystems) according to the manufacturer’s instructions.

#### Allelic imbalance at the rs41284209 *MED4* SNP

To assess a putative *MED4* expression imbalance, cDNAs from 9 patients heterozygous for the rs41284209 *MED4* SNP were analyzed by Sanger sequencing and SNaPshot assay.

In order to avoid contamination by genomic DNA, we amplified a large cDNA fragment (3658 bp) containing the rs41284209 *MED4* SNP and spanning exons 6 to the 3’UTR. Targeted sequencing was then performed using the BigDye Terminator Cycle Sequencing V1.1 Ready Reaction kit (Applied Biosystems) and following electrophoresis in an ABI 3500 Genetic Analyzer (Applied Biosystems). Sequence analyses were performed using Alamut version 2.4 (Interactive Biosoftware, Rouen, France) and FinchTV version 1.4.0 (Geospiza, Inc) softwares. The SNaPshot assay was performed as described below.

#### Allelic imbalance at the c.1981C>T *RB1* mutation

Quantitative SNaPshot assay was performed using primers targeting the c.1981C>T *RB1* mutation and the SNaPshot quantitative primer extension assay (Applied Biosystems), following a previously detailed protocol[26]. Briefly, to determine whether our assay was able to quantitatively measure allelic imbalance, tumor DNA homozygous for the p.Arg661Trp mutation and wild type DNA were mixed at the following ratios (100:0, 80:20, 60:40, 50:50, 40:60, 20:80 and 0:100), then SNaPshot was successfully tested. The peak ratios were measured between the two allelic versions that is, c.1981C to c.1981T/p.Arg661Trp. cDNA ratios were then normalized with respect to the values obtained on genomic DNA (cDNA ratios/gDNA ratios) to correct from putative variations in dye incorporation induced by the nucleotide sequence (see S1 Table for an example). All experiments were performed in duplicate.

## Supporting Information

S1 Table Allelic imbalance within a subset of patients from family F5. (DOC)

S1 Fig *RB1* CpG85 methylation analysis by pyrosequencing in blood samples. The sequence to analyzed is indicated at the top of each pyrogram; Y represents the 9 cytosine residues studied that were either methylated or unmethylated. Bisulfite treatment of DNA converts unmethylated cytosine residues to uracil, whereas 5-methylcytosine residues remain unchanged. Bisulfite-treated DNA sequences will then display a thymine or a cytosine at each CG dinucleotide depending on the methylation status of the cytosine. X axis represents the order of sequential dispensing of enzyme (E), substrate (S) and nucleotides [adenine (A), thymine (T), cytosine (C) and guanine (G)]. Y axis represents peak intensity, which is proportional to the number of dispensed nucleotides incorporated in the sequence. The CG dinucleotides analyzed are shaded on pyrograms. The percentage indicated in colored squares above the corresponding peaks represents the proportion of remaining cytosine residues at the corresponding CG dinucleotide, which in turn indicates the level of methylation at the CG site. The color of the squares above the corresponding peak reflects quality assessment. Yellow represents high quality and blue represents intermediate quality. **A**. Affected *RB1* p.Arg661Trp carrier displaying approximately 50% CpG85 methylation. **B**. Patient with a large deletion of maternal *RB1* allele showing no methylation at CpG85. **C**: Patient with a large deletion of paternal *RB1* allele showing fully methylated CpG85.
(DOC)

S2 Fig Exon 20 RNA analysis. Exon 20 contains the c.1981 C>T mutation. Exon 19/exon 20 junction is indicated at the top of the electrophoregrams. The c.1981C>T mutation is indicated by an arrow. Panel A: without puromycin. Panel B: with puromycin. Non sense mediated decay inhibition by puromycin didn’t reveal any out of frame defect. Targeted RNA analysis showed exon 20 inclusion and absence of skipping.
(TIF)

S3 Fig Methylation analyses of *MED4* CpG53 using methylation array. X axis represents the position on chromosome 13. Y axis represents overall methylation level. *SUCLA2* and *NUDT15* are neighboring genes. *MED4* CpG53 is represented in green. A: Normal retina. CpG53 is unmethylated. B: Tumor sample. CpG53 is unmethylated.
(TIF)

S4 Fig *MED4* expression analysis in lymphocytes analyzed by SNaPshot assay. The *MED4* rs41284209 SNP (c.*783A>G) was used for allelic discrimination. Panel A, genomic results, panel B, cDNA results. No allelic disequilibrium was found.
(TIF)

S5 Fig *MED4* expression analysis in lymphocytes analyzed by Sanger sequencing. The *MED4* rs41284209 SNP (c.*783A>G) was used for allelic discrimination. Electropherograms of 4 heterozygous carriers displayed no allelic disequilibrium on forward (A) and reverse (B) strands.
(TIF)
