# Supplementary material for: A Parent-of-Origin Effect Impacts the Phenotype in Low Penetrance Retinoblastoma Families Segregating the c.1981C>T/p.Arg661Trp Mutation of RB1
Source: PLoS Genet. 2016 Feb 29;12(2):e1005888. doi: 10.1371/journal.pgen.1005888 (PMC4771840; doi:10.1371/journal.pgen.1005888)
Supplement: S1 Table — (DOC) [file pgen.1005888.s001.doc]

Supplemental Table 1. Allelic imbalance within a subset of patients from family F5 (see text for details).

| Patient | Height peak “T”  Mutant allele | | Height peak “C”  Wild type allele | | Ratio “T/C” | | Normalized ratio  cDNA/g.DNA Mutant /Wild type |
| --- | --- | --- | --- | --- | --- | --- | --- |
|  | c.DNA | g.DNA | c.DNA | g.DNA | c.DNA | g.DNA |  |
| II-1 | 7244 | 3502 | 6048 | 5381 | 1.2 | 0.65 | 1.85 |
| II-2 | 7401 | 2746 | 6889 | 3781 | 1.07 | 0.72 | 1.48 |
| III-1 | 1261 | 3007 | 2431 | 5503 | 0.52 | 0.55 | 0.95 |
| III-2 | 1446 | 5119 | 5444 | 7521 | 0.27 | 0.68 | 0.39 |
