# Supplementary material for: A Parent-of-Origin Effect Impacts the Phenotype in Low Penetrance Retinoblastoma Families Segregating the c.1981C>T/p.Arg661Trp Mutation of RB1
Source: PLoS Genet. 2016 Feb 29;12(2):e1005888. doi: 10.1371/journal.pgen.1005888 (PMC4771840; doi:10.1371/journal.pgen.1005888)
Supplement: S1 Fig — The sequence to analyzed is indicated at the top of each pyrogram; Y represents the 9 cytosine residues studied that were either methylated or unmethylated. Bisulfite treatment of DNA converts unmethylated cytosine residues to uracil, whereas 5-methylcytosine residues remain unchanged. Bisulfite-treated DNA sequences will then display a thymine or a cytosine at each CG dinucleotide depending on the methylation status of the cytosine. X axis represents the order of sequential dispensing of enzyme (E), substrate (S) and nucleotides [adenine (A), thymine (T), cytosine (C) and guanine (G)]. Y axis represents peak intensity, which is proportional to the number of dispensed nucleotides incorporated in the sequence. The CG dinucleotides analyzed are shaded on pyrograms. The percentage indicated in colored squares above the corresponding peaks represents the proportion of remaining cytosine residues at the corresponding CG dinucleotide, which in turn indicates the level of methylation at the CG site. The color of the squares above the corresponding peak reflects quality assessment. Yellow represents high quality and blue represents intermediate quality. A. Affected RB1 p.Arg661Trp carrier displaying approximately 50% CpG85 methylation. B. Patient with a large deletion of maternal RB1 allele showing no methylation at CpG85. C: Patient with a large deletion of paternal RB1 allele showing fully methylated CpG85. (DOC) [file pgen.1005888.s002.doc]

**Supplementary figure 1.** *RB1* CpG85 methylation analysis by pyrosequencing in blood samples.

**A.**

**B.**

**C.**
