# Supplementary figures and images for: A Parent-of-Origin Effect Impacts the Phenotype in Low Penetrance Retinoblastoma Families Segregating the c.1981C>T/p.Arg661Trp Mutation of RB1
Source: PLoS Genet. 2016 Feb 29;12(2):e1005888. doi: 10.1371/journal.pgen.1005888 (PMC4771840; doi:10.1371/journal.pgen.1005888)

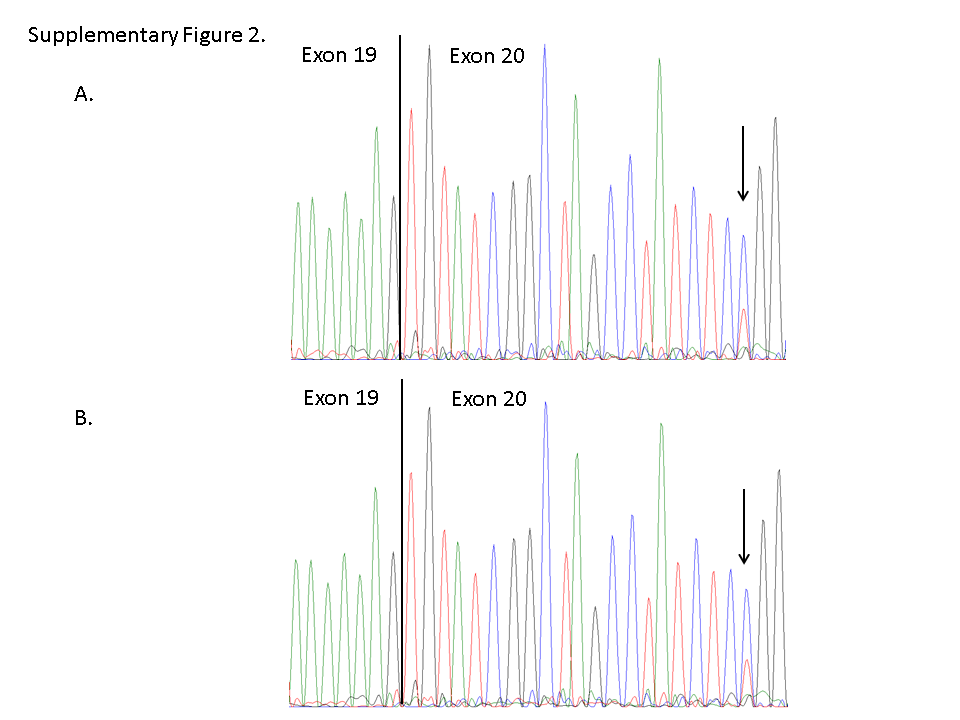

Supplement: S2 Fig — Exon 20 contains the c.1981 C>T mutation. Exon 19/exon 20 junction is indicated at the top of the electrophoregrams. The c.1981C>T mutation is indicated by an arrow. Panel A: without puromycin. Panel B: with puromycin. Non sense mediated decay inhibition by puromycin didn’t reveal any out of frame defect. Targeted RNA analysis showed exon 20 inclusion and absence of skipping. (TIF) [file pgen.1005888.s003.tif]

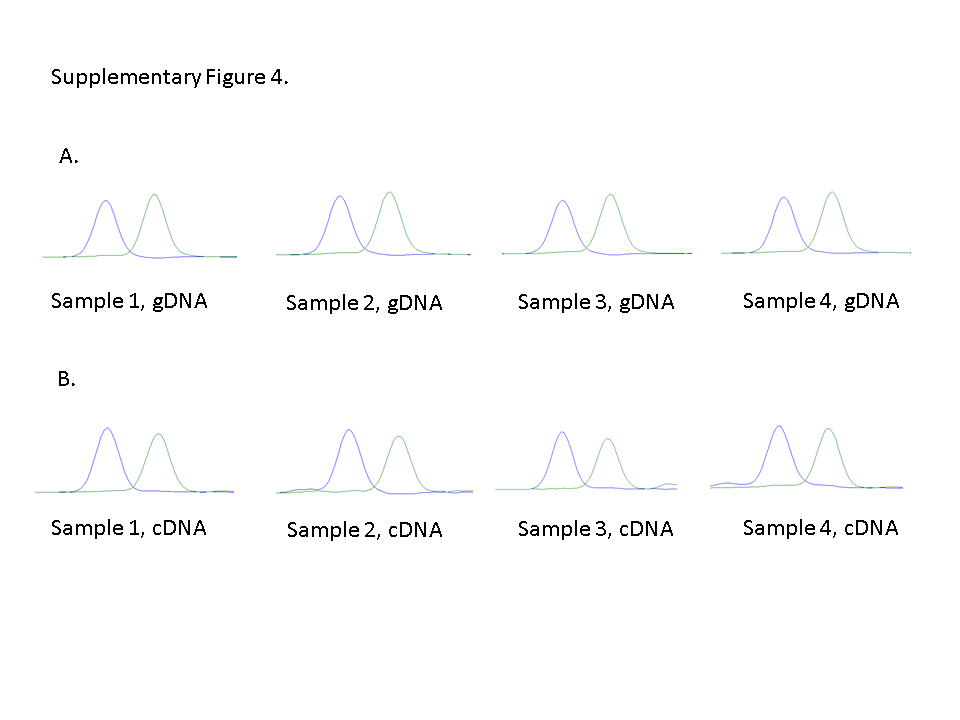

Supplement: S4 Fig — The MED4 rs41284209 SNP (c.*783A>G) was used for allelic discrimination. Panel A, genomic results, panel B, cDNA results. No allelic disequilibrium was found. (TIF) [file pgen.1005888.s005.tif]

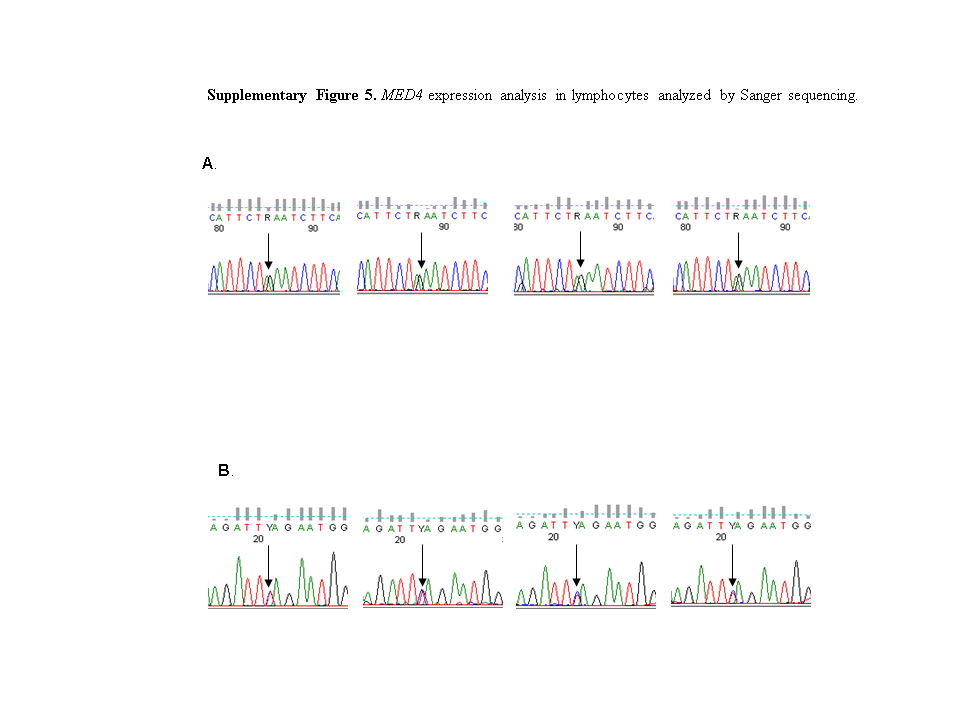

Supplement: S5 Fig — The MED4 rs41284209 SNP (c.*783A>G) was used for allelic discrimination. Electropherograms of 4 heterozygous carriers displayed no allelic disequilibrium on forward (A) and reverse (B) strands. (TIF) [file pgen.1005888.s006.tif]
